# GLP-1RA Liraglutide Attenuates Sepsis by Modulating Gut Microbiota and Associated Metabolites

**DOI:** 10.3390/nu18030531

**Published:** 2026-02-05

**Authors:** Bing Gong, Zhuang’e Shi, Jialong Qi, Fuping Wang, Guobing Chen, Heng Su

**Affiliations:** Faculty of Life Science and Technology, Kunming University of Science and Technology, Kunming 650500, China; 9y121016@kust.edu.cn (B.G.); 20242055@kust.edu.cn (J.Q.);

**Keywords:** sepsis, liraglutide, GLP-1RA, gut microbiota, microbial metabolism, citrulline

## Abstract

Background: Sepsis-induced organ dysfunction poses a significant clinical challenge with limited therapeutic options. This study investigated the therapeutic potential of the glucagon-like peptide-1 receptor agonist (GLP-1RA) liraglutide in sepsis and its underlying mechanisms, focusing on modulation of the gut microbiota-derived metabolome. Methods: Public transcriptomic data analysis identified overlapping targets between liraglutide and sepsis-related genes. In a murine cecal ligation and puncture (CLP) model, liraglutide treatment was evaluated for its effects on survival, systemic inflammation, and organ injury. The gut microbiota composition and fecal metabolome were assessed via 16S rRNA sequencing and UPLC-MS. We also measured plasma GLP-1 in sepsis patients and examined the microbiota-dependency of liraglutide’s effects using antibiotic-depleted mice and fecal microbiota transplantation (FMT) from liraglutide-treated mice. Additionally, citrulline, a key identified metabolite, was functionally validated both in vitro and in a clinical cohort. Results: Liraglutide significantly improved survival, reduced pro-inflammatory cytokines, and alleviated lung, liver, and colon damage in septic mice. It partially restored sepsis-induced gut dysbiosis and modulating associated metabolites, including increasing citrulline. The survival benefit of liraglutide was abolished in microbiota-depleted mice, while FMT from liraglutide-treated mice conferred protection against sepsis, confirming the gut microbiota as a critical mediator. Furthermore, citrulline exhibited direct anti-inflammatory properties in cellular assays, and its plasma levels were negatively correlated with sepsis biomarkers (PCT and CRP) in patients. Conclusions: Taken together, our findings indicate that liraglutide mitigates sepsis by modulating the gut microbiota and regulating associated metabolic pathways. Citrulline may represent a potential microbial mediator or exploratory biomarker within this axis, warranting further mechanistic investigation.

## 1. Introduction

Sepsis is a life-threatening organ dysfunction caused by a dysregulated host response to infection, remains a leading cause of mortality in intensive care units globally [1,2]. Despite advances in antimicrobial therapy and supportive care, treatment options that effectively modulate the host’s maladaptive immune response and protect against multi-organ failure are limited [3,4]. The rising challenge of antimicrobial resistance further underscores the urgent need for novel adjunctive therapies that extend beyond pathogen clearance. In this context, the gastrointestinal tract has emerged as a critical nexus in sepsis pathophysiology. Gut barrier dysfunction and dysbiosis drive a vicious cycle of bacterial translocation and systemic inflammation via the “gut–organ axis,” contributing significantly to distant organ injury and poor outcomes [5,6].

Glucagon-like peptide-1 receptor agonists (GLP-1RAs), initially developed for type 2 diabetes, have demonstrated pleiotropic benefits beyond glycemic control, including anti-inflammatory effects and organ protection in cardiovascular and metabolic diseases [7,8]. Notably, GLP-1RAs can modulate gut motility, barrier integrity, and gut microbiota composition, suggesting a potential role in regulating the gut-centric inflammatory cascade in sepsis [9,10]. However, their potential role in sepsis, particularly through gut-derived mechanisms, remains poorly characterized.

Although GLP-1RAs have been shown to influence both host and microbial metabolism, existing studies have not elucidated whether their protective effects in sepsis involve microbial reprogramming and downstream metabolite production [11,12]. Moreover, it is unclear whether these metabolites could serve as functional mediators with independent therapeutic potential. No studies to date have systematically integrated clinical observations, animal modeling, 16S rRNA sequencing, metabolomics profiling, and functional assays to define the gut microbiota–metabolite axis in the context of sepsis and GLP-1RA treatment.

In this study, we investigated whether liraglutide, a GLP-1RA, confers protection in sepsis via modulation of the gut microbial ecosystem and its metabolic output. Using a cecal ligation and puncture (CLP) mouse model, we evaluated the effects of liraglutide on survival, inflammation, organ injury, microbial composition, and fecal metabolites. To assess the microbiota-dependency of these effects, we employed both antibiotic depletion and fecal microbiota transplantation (FMT) strategies. Finally, we identified citrulline as a representative metabolite enriched following liraglutide treatment, validated its anti-inflammatory potential in vitro, and examined its clinical relevance in a sepsis patient cohort. This integrated approach reveals a candidate microbial–metabolite axis with translational relevance and proposes citrulline as a safer, more defined adjunctive strategy for sepsis therapy.

## 2. Materials and Methods

### 2.1. Clinical Sample Information

This study included a control group (*n* = 29) and a sepsis group (*n* = 39). The control group consisted of individuals with no history of sepsis or trauma. Participants who had used antibiotics or probiotics within the past eight weeks were excluded. Blood samples from 39 sepsis patients were collected within the first three days of admission to the intensive care unit at Yunnan Provincial First People’s Hospital between March and August 2025. All patients who met the inclusion criteria were enrolled consecutively during the study period. Sepsis diagnosis was based on the Sepsis-3 criteria established by the Society of Critical Care Medicine (SCCM) and the European Society of Intensive Care Medicine (ESICM) [13]. Additionally, exclusion criteria included: age under 18, pregnancy, organ transplantation, long-term immunosuppressive therapy, malignancy, hepatitis virus infection, and chronic renal insufficiency. All sepsis patients received standard antibiotic therapy. All experiments were approved by the First People’s Hospital of Yunnan Province Ethics Committee (Project No. KHLL2025-KY164).

### 2.2. Bioinformatics Analysis

Publicly available RNA-sequencing data from the dataset GSE232753 were downloaded from the Gene Expression Omnibus (GEO) database. After quality control and normalization, differential expression analysis between septic and control samples was performed using the limma package in R. Genes with an adjusted *p*-value < 0.05 and absolute log_2_ fold change (|log_2_FC|) > 1 were defined as differentially expressed genes (DEGs). Potential targets of liraglutide were retrieved from the Comparative Toxicogenomics Database (CTD).

### 2.3. Animals and Experimental Design

All experiments were performed in compliance with institutional guidelines for the care and use of animals, and protocols were approved by the Institutional Animal Care and Use Committee (IACUC) at Kunming Medical University (Permission Number: KMMUD2025054). Male C57BL/6J mice, aged 6–8 weeks, were obtained from the Department of Zoology, Kunming Medical University. The mice were housed in a controlled environment with a 12 h light/dark cycle and ad libitum access to water and food.

The number of animals used in this study adhered to the 3Rs principles (Replacement, Reduction, and Refinement) and the required sample sizes for each group were determined using statistical power analysis to ensure that the sample size was adequate for statistical significance. In the liraglutide intervention experiment, the sample sizes for the groups were as follows: Sham (*n* = 10), CLP (*n* = 46), and Lira (*n* = 34). In the antibiotic depletion experiment, the groups included Sham (*n* = 7), ABX (*n* = 29), and CLP (*n* = 20). For the fecal microbiota transplantation (FMT) experiment, each group consisted of 10 animals. All animals underwent an acclimatization period prior to the experiment, and random allocation was performed using a simple randomization method to minimize bias in group assignment. The final sample sizes in each group were sufficient to achieve the desired statistical power, ensuring the reliability of the results.

Lira group: Subcutaneous injections of liraglutide were administered at a dose of 0.2 μg/g to mice, once every 12 h, for 3 consecutive days [14,15]. Following the completion of the treatment regimen, animals were subjected to the CLP-induced sepsis model. ABX+lira group: Antibiotic treatment was administered to mice to deplete the gut microbiota prior to the induction of sepsis. The antibiotic cocktail consisted of ampicillin (200 mg/kg), vancomycin (100 mg/kg), neomycin (200 mg/kg), and metronidazole (200 mg/kg), with gavage once daily for 5 days to ensure a thorough depletion of the microbiota [2], before the CLP animals were treated with liraglutide as described above. The surgical procedures were carried out under general anesthesia with nembutal, and mice were monitored for survival daily for 7 days. Survival was analyzed using the log-rank (Mantel–Cox) test.

Fecal microbiota transplantation (FMT): Fecal pellets were freshly collected from donor mice (CLP and Lira groups, as described in Section 2.3) under sterile conditions. The fecal material was homogenized in sterile phosphate-buffered saline (PBS) at a concentration of 30 mg/mL, followed by a brief centrifugation (1000 rpm for 5 min) to remove large particulate matter [16]. The supernatant (fecal slurry) was then used for gavage within 15 min of preparation. Recipient mice were pretreated with a 5-day regimen of broad-spectrum antibiotics (as described in Section 2.3) to deplete their native gut microbiota. After the final antibiotic dose, each receptor mice was gavaged with 200 μL of the prepared fecal slurry for 5 days, after that the CLP-induced sepsis model was established. The survival of recipient mice was monitored daily for 7 days post-CLP. The mice were euthanized at 16 h after CLP induction to obtain serum and tissues.

### 2.4. Histopathological Examination

At the end of the experimental period, animals were euthanized, and tissues from the lung, liver, colon, and kidney were harvested for histopathological analysis. Tissues were fixed in 10% formalin, embedded in paraffin, and sectioned at 5 μm thickness. Sections were stained with hematoxylin and eosin (H&E) and evaluated for histological changes such as inflammation, edema, and tissue damage. Tissue damage was scored on a scale from 0 (no damage) to 4 (severe damage) by a blinded pathologist. The histopathological evaluation of the colon, lung, and liver tissues was conducted according to established scoring criteria (3–5). The histopathological evaluation of the colon, lung, and liver tissues was conducted according to established scoring criteria.

### 2.5. Cytokine and Inflammatory Mediator Measurement

Serum and bronchoalveolar lavage fluid (BALF) and peritoneal lavage fluid (PLF) were collected for cytokine analysis. The levels of TNF-α, IL-1β, and IL-6 were quantified using commercially available ELISA kits (KEQIAOBIO, Shanghai, China) following the manufacturer’s instructions. The concentrations of cytokines were calculated from standard curves and expressed as pg/mL.

### 2.6. Microbiota Analysis

Fecal samples were collected at the end of the study for microbiota analysis. The V3-V4 region of the 16S rRNA gene was amplified and sequenced on the Illumina MiSeq v2 platform. Sequences were analyzed using QIIME2 (version 2024.2) to evaluate alpha diversity (Shannon and Chao1 indices) and beta diversity (Bray–Curtis dissimilarity). Principal Coordinate Analysis (PCoA) was used to visualize group differences. Differentially abundant taxa were identified using LEfSe (Linear Discriminant Analysis Effect Size), with an LDA threshold of 4.0 and a *p*-value cutoff of 0.05.

### 2.7. Metabolomic Analysis

Metabolite profiling was performed on fecal samples from all groups to assess metabolic alterations induced by sepsis and liraglutide treatment. Metabolomics profiling was analyzed using a UPLC-ESI-Q-Orbitrap-MS system (UHPLC, Shimadzu Nexera X2 LC-30AD, Shimadzu, Japan) coupled with Q-Exactive Plus (Thermo Scientific, San Jose, CA, USA).

For liquid chromatography (LC) separation, samples were analyzed using an ACQUITY UPLC^®^ HSS T3 column (2.1 × 100 mm, 1.8 μm) (Waters, Milford, MA, USA). The flow rate was 0.3 mL/min and the mobile phase contained: A, 0.1% FA in water; B, 100% acetonitrile (ACN). The gradient was 0% buffer B for 2 min and was linearly increased to 48% in 4 min, and then up to 100% in 4 min and maintained for 2 min, and then decreased to 0% buffer B in 0.1 min, with a 3 min re-equilibration period employed.

Following normalization and scaling, differential metabolites were identified using univariate statistical tests (*t*-test). To control for multiple comparisons, the resulting *p*-values were adjusted using the Benjamini–Hochberg false discovery rate (FDR) correction. Metabolites with an FDR-adjusted *p*-value (q-value) < 0.05 and a fold-change ∣FC∣ ≥ 1.5 were considered statistically significant.

### 2.8. Targeted Quantification of Circulating Citrulline

Human plasma and mouse feces citrulline concentrations were determined using a validated liquid chromatography–tandem mass spectrometry (LC-MS/MS) method. Briefly, 100 μL of plasma was diluted with 900 μL of ice-cold extraction solvent (50% methanol/50% acetonitrile, *v*/*v*). The mixture was vortexed, sonicated in an ice bath for 10 min, and centrifuged at 13,000× *g* for 10 min at 4 °C. The resulting supernatant was collected for analysis. Chromatographic separation was achieved on a Shimadzu LC-30AD UPLC system using a HILIC column (2.1 × 150 mm, Waters, Milford, MA, USA). The mobile phase consisted of (A) 0.1% formic acid in water and (B) 0.1% formic acid in acetonitrile, with a gradient elution at a flow rate of 0.3 mL/min. Mass spectrometric detection was performed on a SCIEX QTRAP 6500+ instrument (SCIEX, Marlborough, MA, USA) equipped with an electrospray ionization source operating in positive mode. Quantification was based on a six-point external standard calibration curve (0.1–10 μmol/L), which showed excellent linearity (R^2^ > 0.997). Method reliability was confirmed by quality control (QC) samples injected throughout the analytical batch, demonstrating a coefficient of variation (CV) of 4.98% and a recovery rate of 130.7%.

### 2.9. Cell Culture and Citrulline Treatment

Two murine cell lines were employed: the RAW264.7 macrophage cell line and the MLE-12 lung epithelial cell line. RAW264.7 cells were maintained in Dulbecco’s Modified Eagle Medium (DMEM), while MLE-12 cells were maintained in a 1:1 mixture of DMEM and Ham’s F-12 medium (DMEM/F-12). All media were supplemented with 10% heat-inactivated fetal bovine serum, and the cells were cultured at 37 °C in a humidified atmosphere containing 5% CO_2_.

To assess the immunomodulatory capacity of citrulline, the cells were seeded in plates and allowed to adhere overnight. Based on preliminary experiments and the existing literature, the cells were pretreated with 500 μM citrulline or an equal volume of the vehicle (PBS) for 4 h. Subsequently, the pretreated cells were directly stimulated by adding ultrapure LPS (Sigma L2880) to the existing culture medium at a final concentration of 0.2 μg/mL (RAW 264.7) or 1 μg/mL (MLE-12) [17,18]. After 20 h of LPS stimulation, the cells were harvested for subsequent analysis.

### 2.10. Statistical Analysis

All data are presented as the mean ± SEM. For comparisons between two groups, an unpaired Student’s *t*-test was used. For comparisons among more than two groups, one-way analysis of variance (ANOVA) followed by Tukey’s post hoc test was performed. Survival data were analyzed using the log-rank (Mantel–Cox) test. A *p*-value of < 0.05 was considered statistically significant. For metabolomics analyses, *p*-values were adjusted for multiple testing using the Benjamini–Hochberg procedure to control the false discovery rate (FDR). A q-value < 0.05 was considered significant. All statistical analyses were conducted using GraphPad Prism version 9.5 and R (version: 4.5.1).

## 3. Results

### 3.1. GLP-1RA Liraglutide Targets Overlap with Sepsis-Related Inflammatory Mediators

**Figure 1:**
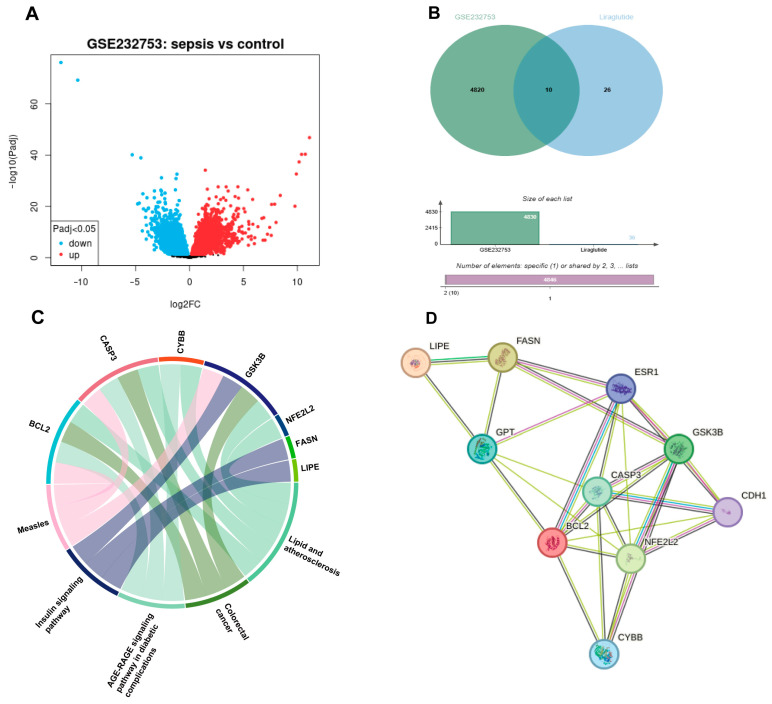
Integrated bioinformatics and clinical validation underscore the potential role of GLP-1 receptor agonism in sepsis. (**A**) Volcano plot of DEGs in septic versus control samples. (**B**) Overlap between sepsis-related DEGs and predicted liraglutide targets. (**C**) Pathway mapping of overlapping genes. (**D**) PPI network of core targets.

We analyzed the publicly available transcriptome dataset GSE232753 to characterize the gene expression changes associated with sepsis. Differential expression analysis (cut-off: *p* < 0.05, |log2FC| > 1) revealed a large number of upregulated and downregulated genes in septic samples compared with controls (Figure 1A). Transcriptomic profiling reveals differentially expressed genes in sepsis.

To explore the potential molecular mechanisms underlying liraglutide’s protective effects in sepsis, we first retrieved 36 candidate targets of liraglutide from the CTD database, including well-established regulators such as GLP1R, IL1B, TNF, IL6, BAX, BCL2, CASP3, and AKT1. These targets are mainly enriched in inflammation, apoptosis, oxidative stress, and metabolic regulation (Figure 1B). The Venn diagram shows the overlap between sepsis-related DEGs and predicted liraglutide targets, with 10 overlapping genes identified.

Functional analysis of the overlapping genes indicated that liraglutide’s targets span multiple pathological pathways. A chord diagram illustrated that these genes (for example, BCL2, CASP3, LIPE, and FASN) are enriched in processes related to inflammation, apoptosis, oxidative stress, and metabolic regulation (Figure 1C). Similarly, a protein–protein interaction (PPI) network showed that liraglutide targets interact with central nodes of inflammatory and apoptotic signaling (Figure 1D), highlighting the drug’s potential to modulate key aspects of sepsis pathophysiology.

### 3.2. GLP-1RA Liraglutide Improves Survival and Reduces Inflammation in Septic Mice

**Figure 2:**
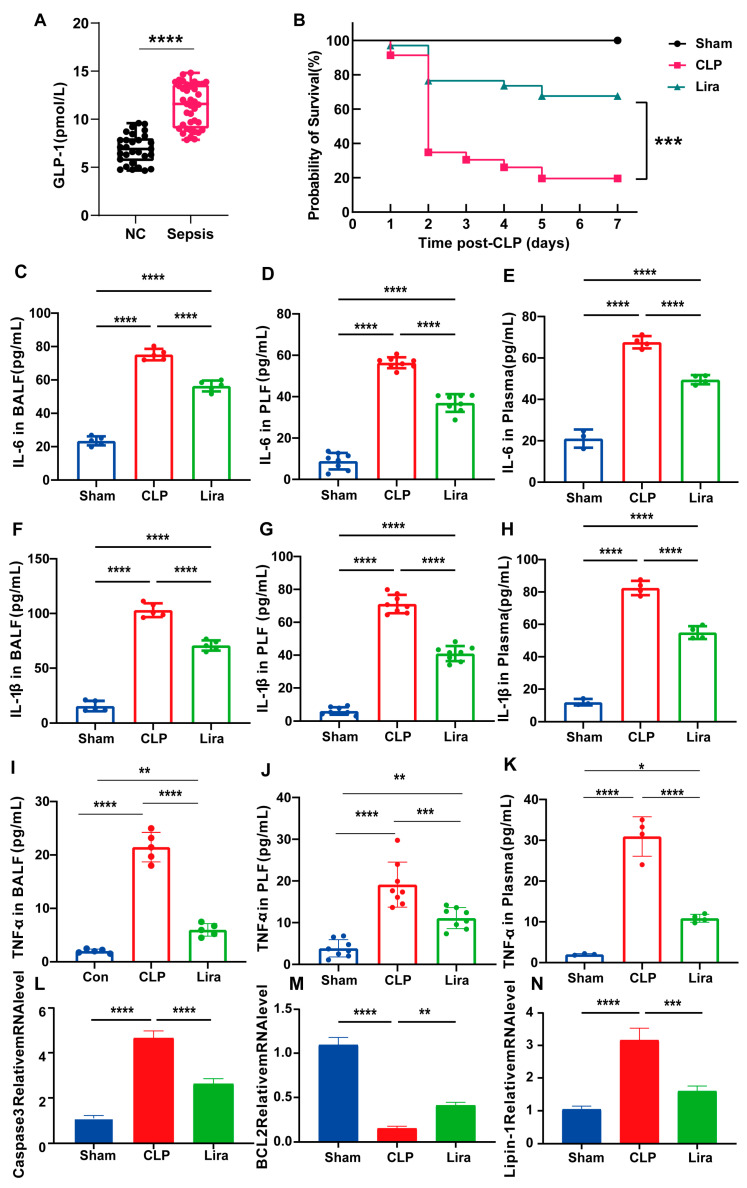
Liraglutide supplementation improves survival and reduces inflammation in septic mice. (**A**) Plasma GLP-1 concentrations were significantly elevated in patients with sepsis (*n* = 39) compared to healthy controls (*n* = 29). (**B**) Kaplan–Meier survival curves of CLP-induced septic mice with or without liraglutide treatment (Sham, *n* = 10; CLP, *n* = 46; liraglutide, *n* = 34). (**C**–**E**) The levels of IL-6 in serum (Sham, *n* = 3; CLP, *n* = 4; Lira, *n* = 4) BALF (*n* = 5/group) and PLF (*n* = 8/group). (**F**–**H**) The levels of IL-1β in serum, BALF and PLF. (**I**–**K**) The levels of TNF-α in serum, BALF and PLF. (**L**–**N**) Relative mRNA expression of *Caspase-3*, *BCL2*, and *Lipin-1* in lung tissues. (* *p*  <  0.05, ** *p*  <  0.01, *** *p*  <  0.001, **** *p*  <  0.0001).

To assess the clinical relevance of the GLP-1 pathway in human sepsis, we first measured plasma GLP-1 levels in septic patients (*n* = 39) and healthy controls (*n* = 29). GLP-1 concentrations were significantly elevated in the sepsis group, suggesting endogenous activation of this axis during systemic inflammation (Figure 2A). This elevation, however, may not fully reflect functional GLP-1 receptor signaling, supporting the rationale for augmenting the pathway with exogenous GLP-1R agonists.

Next, we investigated the protective effect of liraglutide in a murine CLP model. The therapeutic relevance of GLP-1RA liraglutide was next evaluated in vivo. Administration of liraglutide significantly improved outcomes in septic mice subjected to cecal ligation and puncture (CLP). Survival analysis demonstrated that liraglutide-treated animals exhibited a markedly higher survival rate compared with untreated septic controls, indicating a protective effect against sepsis-induced lethality (Figure 2B). This survival benefit was accompanied by marked attenuation of pro-inflammatory cytokines across multiple compartments. Specifically, liraglutide reduced IL-6, IL-1β, and TNF-α levels in bronchoalveolar lavage fluid (BALF), peritoneal lavage fluid (PLF), and plasma (Figure 2C–K), indicating systemic anti-inflammatory effects.

Furthermore, liraglutide modulated the expression of key sepsis-related transcriptional regulators in lung tissue, thereby functionally validating targets identified in the transcriptomic analysis. Specifically, in septic mice, both *Caspase-3* and *Lipin-1* were significantly upregulated, while the anti-apoptotic gene *BCL2* was downregulated, reflecting enhanced apoptosis and dysregulated metabolic responses. Liraglutide treatment reversed these alterations: *Caspase-3* and *Lipin-1* expression levels were significantly reduced, and *BCL2* was restored (Figure 2L–N). These findings confirm the in vivo relevance of the transcriptomic predictions and suggest that liraglutide modulates not only inflammatory but also apoptotic and metabolic signaling pathways during sepsis.

### 3.3. Liraglutide Treatment Ameliorates Organ Damage in Septic Mice

**Figure 3:**
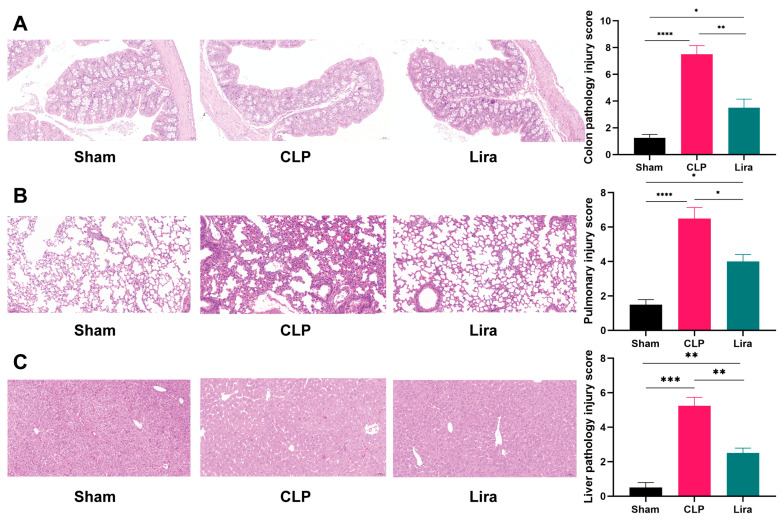
Protective effects of liraglutide on organ injury in septic mice. (**A**) Histology and injury score of colon tissue. (**B**) Histology and injury score of lung tissue. (**C**) Histology and injury score of liver tissue (H&E staining, 20.0×; scale bars: 50 μm; *n* = 4, respectively). (* *p*  <  0.05, ** *p*  <  0.01, *** *p*  <  0.001, **** *p*  <  0.0001).

Histological examination of colon tissue demonstrated severe structural disruption in septic mice. The mucosal layer was markedly thinned, with disorganized and partially atrophic or absent crypt glands. Goblet cell numbers were reduced, and epithelial cells exhibited varying degrees of detachment and necrosis. The lamina propria and submucosa showed massive inflammatory cell infiltration accompanied by vascular dilatation and interstitial edema. Smooth muscle cells in the muscular layer displayed hydropic swelling, with cytoplasmic rarefaction and occasional vacuolar degeneration, indicating impaired barrier function. In contrast, liraglutide treatment markedly alleviated these pathological changes. The mucosal architecture appeared relatively preserved, crypt glands were more regularly arranged, and the number of goblet cells was significantly increased compared with the sepsis group. Epithelial cell loss was reduced, and inflammatory infiltration in the lamina propria and submucosa was substantially decreased, along with attenuated vascular dilatation and edema. Moreover, smooth muscle swelling and cytoplasmic vacuolization were less pronounced, suggesting a protective effect of liraglutide on colonic barrier function in CLP mice (Figure 3A).

Similarly, histopathological analysis of the lungs showed extensive alveolar destruction, interstitial edema, and inflammatory infiltration in septic mice. However, liraglutide treatment helped to preserve alveolar structures, reducing the severity of pathological changes. The lungs of liraglutide-treated mice exhibited less alveolar destruction and a marked reduction in inflammatory infiltration, further supporting the protective role of liraglutide against lung injury. Lung injury scores also showed significant improvement with liraglutide treatment (Figure 3B).

In addition to the gut and lungs, liver sections from septic mice revealed vacuolar and hydropic degeneration of hepatocytes, with cytoplasmic rarefaction and occasional nuclear displacement. The normal cord-like arrangement of hepatocytes was disrupted, and sinusoidal spaces were dilated and congested with red blood cells. However, liraglutide treatment markedly alleviated these pathological changes, preserving hepatic architecture, reducing hepatocyte degeneration, and attenuating sinusoidal congestion. The liver pathology score also showed significant improvement, further demonstrating the beneficial effects of liraglutide on multiple organs (Figure 3C). These results collectively highlight the protective effects of liraglutide on various organs in septic mice, suggesting its potential as a therapeutic intervention for sepsis-induced organ damage.

### 3.4. Liraglutide Reshapes Gut Microbial Diversity and Composition

Alpha diversity indices (Chao1 and Shannon, Simpson and Pielou’s index) of gut microbiota in Sham, CLP, and Lira groups. Sepsis markedly reduced microbial diversity, which was partially restored by liraglutide treatment.

**Figure 4:**
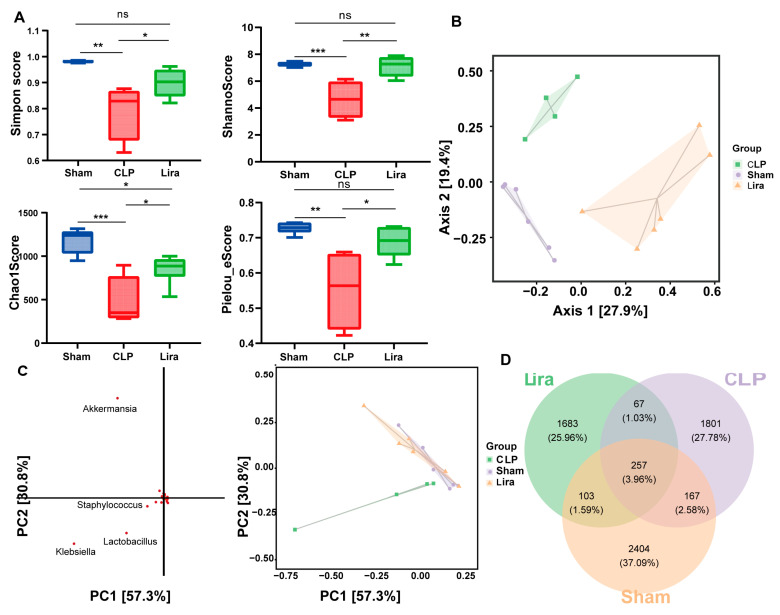
Liraglutide modulates gut microbial diversity and community structure in septic mice. (**A**) α-diversity indices. (**B**) Principal coordinate analysis (PCoA) based on Bray–Curtis distances shows distinct clustering of the three groups. (**C**) Principal component analysis (PCA) at the genus level. Key genera contributing to group separation are indicated (e.g., *Klebsiella*, *Lactobacillus*, *Akkermansia*). (**D**) Venn diagram. (ns, not significant, * *p* < 0.05, ** *p* < 0.01, *** *p* < 0.001, *n* = 4–6).

To investigate whether liraglutide affects the gut microbiota, we assessed microbial α-diversity by analyzing richness (Chao1), diversity (Shannon and Simpson indices, reported as 1–D), and evenness (Pielou’s index). Sepsis (CLP) significantly reduced richness, diversity, and evenness, while liraglutide treatment partially restored these metrics towards control levels (Figure 4A). The Chao1 index was significantly lower in the CLP group than in the control group (*** *p* < 0.001), indicating a reduction in species richness. Liraglutide + CLP mice showed a higher Chao1 value compared with the CLP group (* *p* < 0.05) but remained below the control level (* *p* < 0.05). The Shannon index (Figure 4B) was also reduced in the CLP group compared with controls (*** *p* < 0.001). Liraglutide treatment increased Shannon diversity relative to CLP (** *p* < 0.01), showing no significant difference from controls. Similarly, the Simpson index (1–D) and Pielou’s evenness were both significantly decreased in the CLP group (** *p* < 0.01) and elevated in the liraglutide + CLP group compared with CLP alone (* *p* < 0.05), with no difference relative to controls.

Collectively, these findings demonstrate that sepsis leads to a marked loss of microbial richness, diversity, and evenness, while liraglutide treatment mitigates these changes and restores gut microbial community balance.

Beta diversity was assessed using abundance-weighted Bray–Curtis dissimilarity and visualized through PCoA (Figure 4B). The PERMANOVA analysis revealed significant compositional separation among the Sham, CLP and liraglutide-treated (Lira) groups (F = 4.54, R^2^ = 0.411, *p* = 0.001), indicating that sepsis induces a substantial structural shift in the gut microbiota. Axis 1 and Axis 2 of the PCoA ordination explained 27.9% and 19.4% of the variance, respectively. The Lira group clustered on the positive side of Axis 1, distinctly separated from both the CLP and the Sham groups. The CLP group and Sham group were further differentiated primarily along Axis 2.

Principal component analysis (PCA) based on genus-level relative abundances was performed to visualize compositional variation among groups (Figure 4C). PC1 and PC2 explained 57.3% and 30.8% of the total variance, respectively. In the loading plot, *Klebsiella* and *Lactobacillus* showed negative loadings on PC1, corresponding to the C group direction, while *Akkermansia* had a negative loading on PC1 and a positive loading on PC2, contributing mainly to the separation of Lira.

A Venn diagram provided an overview of shared richness among groups. In total, 6482 ASVs/OTUs were detected, of which only 257 (3.96%) were shared by all three groups (Figure 4D). Each group also contained a large set of unique taxa, with Sham showing the most group-specific ASVs/OTUs (*n* = 2404), followed by CLP (*n* = 1801) and Lira (*n* = 1683). These data indicate limited overlap among groups and a lower unique richness in CLP and Lira relative to Sham. Collectively, the data indicate that liraglutide mitigates CLP-induced dysbiosis, partially restores gut microbial diversity and community structure, and modulates key microbial genera, supporting its role in ameliorating sepsis-associated gut dysfunction.

### 3.5. Liraglutide Modulates the Gut Microbiota Composition in Septic Mice

**Figure 5:**
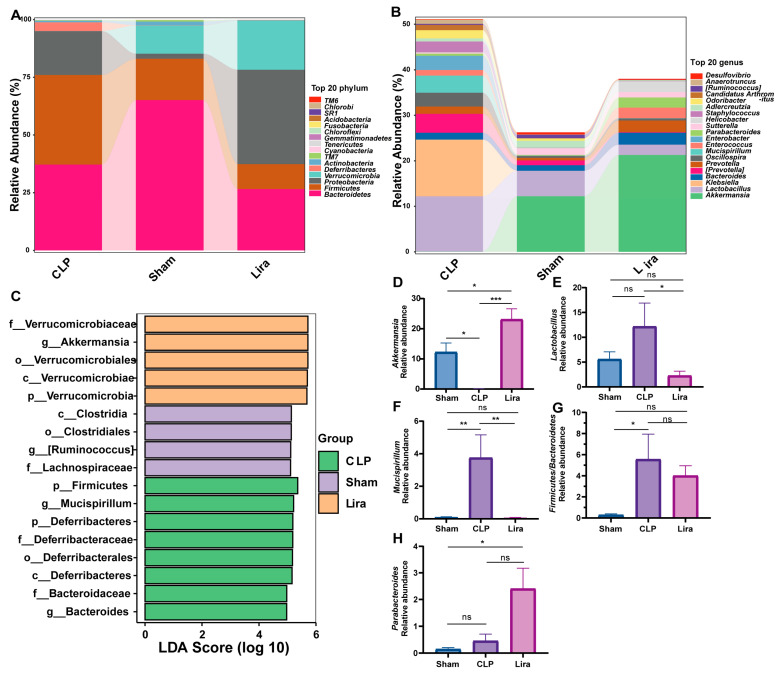
Liraglutide modulates the gut microbiota composition in septic mice. (**A**) Relative abundance of the dominant bacterial phyla across Sham, CLP, and liraglutide (Lira)-treated groups. (**B**) Heatmap showing the relative abundance of the top 20 bacterial genera. (**C**) LEfSe analysis (LDA score > 4) identifying taxonomic clades differentially abundant among groups. The CLP group shows enrichment of *Firmicutes* and *Actinomycetales*. The Lira group is characterized by a distinct *Verrucomicrobia*–*Akkermansia* lineage. (**D**–**H**) Bar plots comparing the relative abundances of selected genera (*Akkermansia*, *Lactobacillus*, and *Mucispirillum*) and the *Firmicutes*/*Bacteroidetes* (F/B) ratio across groups. Data are presented as the mean ± SEM. Statistical significance was determined by one-way ANOVA followed by post hoc tests (* *p* < 0.05, ** *p* < 0.01, *** *p* < 0.001; ns, not significant; *n* = 4–6 per group).

Analysis of the gut microbiota across the three groups showed clear differences at the phylum level (Figure 5A). The Sham group was dominated by *Bacteroidetes*, whereas *Firmicutes* and *Proteobacteria* accounted for lower proportions. The CLP group exhibited a marked increase in *Firmicutes* with a concomitant decrease in *Bacteroidetes*. The Lira group showed an expansion of *Proteobacteria* accompanied by an increase in Verrucomicrobia. Consistently, compared with the Sham, the CLP group showed an increase in the *Firmicutes*-to-*Bacteroidetes* (F/B) ratio, these shifts were partially attenuated after liraglutide (GLP-1RA) treatment.

At the genus level, we visualized the relative abundance of the top 20 dominant genera across the three groups (Figure 5B). This analysis revealed dramatic shifts in the gut microbial community structure, most notably involving the genera *Klebsiella* and *Akkermansia*. A striking feature of the CLP group was a dramatic bloom of *Klebsiella*, which became one of the most dominant genera in this group. In stark contrast, the relative abundance of *Klebsiella* was negligible in both the control (Sham) and the liraglutide-treated (Lira) groups. Concurrently, the abundance of *Akkermansia*, an emerging beneficial bacterium, was severely depleted to nearly undetectable levels in the CLP group compared to its substantial presence in the controls. Remarkably, following liraglutide administration, the Lira group exhibited a robust and significant increase in the relative abundance of *Akkermansia*, with levels surpassing those observed in the healthy control group.

LEfSe results (LDA > 4, *p* < 0.05) identifying discriminatory taxa among Sham, CLP, and Lira. Sham is enriched for SCFA-related clades (*Clostridia*/*Clostridiales*/*Lachnospiraceae* and *[Ruminococcus]*). CLP shows higher-rank enrichment of *Firmicutes* and *Actinomycetales*. Lira displays a phylum-to-genus *Verrucomicrobia*–*Akkermansia* lineage (*Verrucomicrobia* → *Verrucomicrobiae* → *Verrucomicrobiales* → *Verrucomicrobiaceae* → *Akkermansia*) (Figure 5C).

To identify specific genera driving the observed community shifts, we compared the relative abundances of several key bacteria, including *Akkermansia*, *Lactobacillus*, and *Mucispirillum*, across the experimental groups. The relative abundance of *Akkermansia* was significantly depleted in the CLP group compared to the control (Sham) group (* *p* < 0.05). Remarkably, liraglutide treatment not only reversed this depletion but robustly elevated the abundance of *Akkermansia* to a level significantly higher than that of both the CLP group (*** *p* < 0.001) and the healthy controls (* *p* < 0.05). In contrast, the relative abundance of *Lactobacillus* was elevated in the CLP group, although this difference did not reach statistical significance compared with the controls (ns). Liraglutide administration significantly reduced *Lactobacillus* abundance relative to the CLP group (* *p* < 0.05), restoring it to a level comparable to that of the control group (ns). The CLP group showed a marked increase in the relative abundance of *Mucispirillum* compared with controls (** *p* < 0.01).

Liraglutide treatment significantly reduced *Mucispirillum* relative to CLP (** *p* < 0.01), but it was not significantly different from controls (ns). Similarly, compared with controls, the CLP group exhibited an increased *Firmicutes*-to-*Bacteroidetes* (F/B) ratio and a higher relative abundance of Lactobacillus. Treatment with liraglutide (GLP-1 RA) showed a trend toward reduction in both indices relative to CLP, although the differences did not reach statistical significance (Figure 5D–H).

### 3.6. Liraglutide Reprograms the Gut Microbial Metabolites in Septic Mice

**Figure 6:**
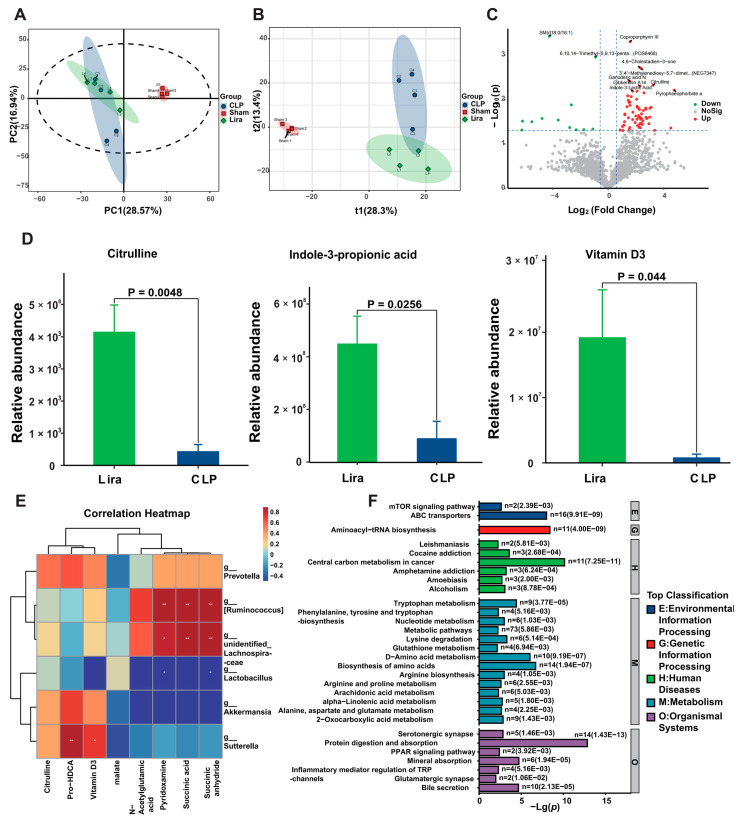
Liraglutide remodels the gut microbial metabolomic profile in septic mice. (**A**) Principal component analysis (PCA) scores plot of fecal metabolites. (**B**) Partial least squares-discriminant analysis (PLS-DA) scores plot demonstrating clear metabolic separation between CLP and Lira groups (t1 = 28.3%, t2 = 13.4%). (**C**) Volcano plot displaying metabolites differentially abundant between CLP and Lira groups. (**D**) Box plots showing the abundance of key individual metabolites significantly altered by liraglutide treatment (q-value < 0.05). (**E**) Spearman correlation heatmap between differentially abundant microbial genera and metabolites. Color intensity indicates the strength of the correlation, * *p* < 0.05, ** *p* < 0.01. (**F**) KEGG pathway enrichment analysis of the differential metabolites. The top 30 enriched pathways are displayed, ranked by enrichment significance.

We next performed untargeted metabolomic profiling on fecal samples to determine whether liraglutide-induced microbial remodeling was accompanied by functional changes in metabolic output. Principal component analysis (PCA) revealed a clear separation in the overall metabolic profiles among the Sham, CLP, and Lira groups, indicating profound sepsis-induced metabolic reprogramming that was partially shifted by liraglutide treatment (Figure 6A). The first principal component (PC1, 28.57%) and the second principal component (PC2, 16.94%) explain the majority of the variance in the dataset, with a cross-validated predictability of Q2 (0.443), indicating a stable model.

Supervised partial least squares-discriminant analysis (PLS-DA) further confirmed a distinct metabolic signature in liraglutide-treated mice compared to CLP controls (Figure 6B). The two groups were clearly separated along the first two principal components (t1 = 28.3%, t2 = 13.4%), indicating a distinct metabolic shift following liraglutide treatment. These results suggest that liraglutide induced a marked remodeling of the gut metabolic profile, consistent with the compositional changes observed in the microbiota analysis.

Comparative analysis identified 75 metabolites significantly altered by liraglutide treatment (fold change ≥ 1.5 or ≤ 1/1.5, *p* < 0.05), with 63 upregulated and 12 downregulated (Figure 6C). A clustered heatmap of these differential metabolites distinctly separated the Lira group from the CLP group. The heatmap annotations further indicate that the responsive metabolites are primarily involved in steroid hormone metabolism, amino acids and derivatives glycerophospholipid remodeling, and tryptophan/indole metabolism. (Supplementary Figure S1). Notably, among the upregulated metabolites were Indole-3-Lactic Acid, Citrulline, and Ganoderic acid N. Red dots on the plot represent upregulated metabolites, while blue dots indicate downregulated metabolites, as identified by univariate statistical analysis.

To further characterize specific metabolites altered by liraglutide, we quantified representative differential compounds with known biological relevance. Among them, citrulline, indole-3-propionic acid, and vitamin D_3_ were significantly elevated in the Lira group compared to CLP controls (Figure 6D). These metabolites have been previously implicated in gut barrier function, anti-inflammatory signaling, and host–microbe metabolic integration. While their elevation aligns with improved outcomes in Lira-treated mice, this association remains correlative, and further mechanistic validation is warranted. The levels of Artemisinin, ProstaglandiZn E2 and Grandiflorenic Acid were increased. Concurrently, reductions were observed in PC(16:1/16:1) and PC(16:0/22:6). These data demonstrate that liraglutide treatment is associated with distinct changes in metabolites involved in amino acid metabolism, lipid species, and compounds of dietary or microbial origin (Supplementary Figure S2).

We next performed a Spearman correlation analysis between altered microbial genera and differential metabolites. As shown in the correlation heatmap (Figure 6E), genera such as *Prevotella*, *Ruminococcus*, and *Akkermansia* exhibited trend-level positive associations with beneficial metabolites including citrulline and vitamin D_3_. However, none of these correlations reached statistical significance, and they should be interpreted as exploratory indicators of co-variation rather than direct functional interactions. Furthermore, enrichment analysis (KEGG) of the differential metabolites identified significant perturbations in pathways related to amino acid biosynthesis and metabolism (e.g., tryptophan metabolism), nutrient transport (ABC transporters), and immunometabolic regulation, including arachidonic acid metabolism and PPAR signaling (Figure 6F). These results indicate that liraglutide remodels key host–microbiota metabolic networks involved in nutrient processing and inflammatory responses during sepsis.

### 3.7. Protective Effects of GLP-1RA Require the Gut Microbiota

Given that liraglutide modulated several gut microbiota-associated metabolites as described above, we hypothesized that some of its beneficial effects might be dependent on an intact gut microbiota. To test this hypothesis, we re-evaluated the efficacy of liraglutide in CLP mice following depletion of the gut microbiota with a broad-spectrum antibiotic cocktail (ABX).

**Figure 7:**
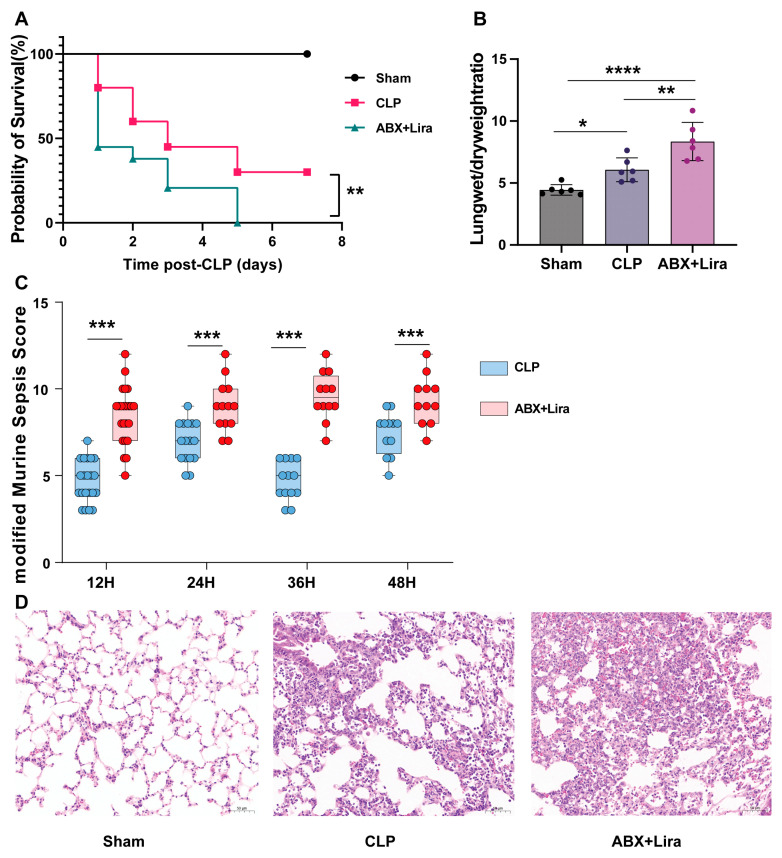
The protective effects of liraglutide in sepsis are dependent on an intact gut microbiota. (**A**) Kaplan–Meier survival curves of CLP-induced septic mice pretreated with a broad-spectrum antibiotic cocktail (ABX) to deplete the gut microbiota, with or without liraglutide treatment (Sham, *n* = 7; ABX, *n* = 29; CLP, *n* = 20). (**B**) Modified sepsis severity scores. (**C**) Assessment of pulmonary edema (*n*  =  6/group). (**D**) Representative lung histology (H&E staining) and injury scores demonstrating more severe pathological damage in ABX-pretreated CLP mice. (bar  =  50 μm; *n*  =  3/group). Data are presented as the mean ± SEM. Statistical analyses were performed using one-way ANOVA or the log-rank test (survival) (* *p*  <  0.05, ** *p*  <  0.01, *** *p*  <  0.001, **** *p*  <  0.0001); *n* values are indicated in the figure or corresponding section).

Antibiotic-mediated gut microbiota depletion not only abolished the therapeutic effect of liraglutide but also exacerbated sepsis mortality (Figure 7A), indicating a protective role of the commensal microbiota against sepsis. As previously observed, liraglutide treatment significantly improved survival in microbiota-intact mice. However, liraglutide completely failed to improve survival in microbiota-depleted mice (ABX + Lira).

Consistent with the survival outcome, ABX pretreatment aggravated organ injury, as evidenced by significantly higher modified sepsis severity scores (Figure 7B), leading to more severe pulmonary edema (Figure 7C) and more pronounced histopathological damage in the lungs compared to the CLP group (Figure 7D).

### 3.8. Fecal Microbiota Transplantation from Liraglutide-Treated Mice Partially Restores Protection Against Sepsis

**Figure 8:**
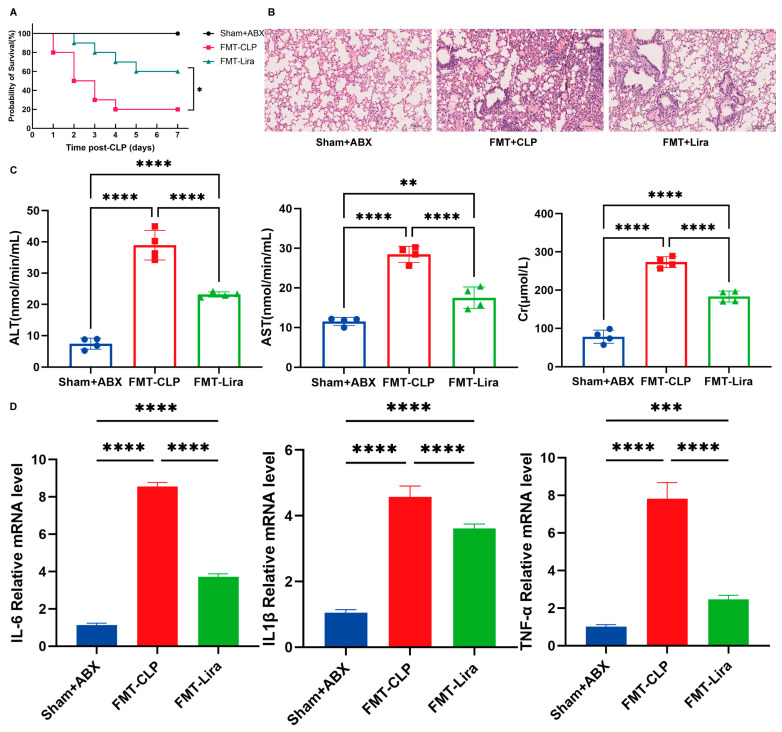
Fecal microbiota transplantation (FMT) restores protective effects of liraglutide in septic mice. (**A**) Survival curves of CLP mice receiving FMT from liraglutide-treated (FMT-Lira) or untreated (FMT-CLP) donor septic mice, compared to untreated ABX controls (Sham + ABX). Liraglutide-induced protection was partially restored in FMT-Lira mice (*n* = 10/group). (**B**) Histological analysis of lung tissue showing reduced damage in FMT-Lira mice compared to FMT-CLP controls (20.0×; scale bars 20 μm). (**C**) Serum markers of liver and kidney injury (ALT, AST, and creatinine) in FMT-Lira and FMT-CLP mice. FMT-Lira recipients exhibited lower levels of these markers, indicating reduced organ injury (*n* = 4/group). (**D**) Inflammatory cytokine levels (IL-6, TNF-α, and IL-1β) in the lung tissue of FMT-Lira and FMT-CLP mice. Cytokine levels were significantly reduced in FMT-Lira mice, suggesting attenuation of systemic inflammation (*n* = 4/group). * *p*  <  0.05, ** *p*  <  0.01, *** *p*  <  0.001, **** *p*  <  0.0001.

To determine whether the protective effects of liraglutide are mediated through the gut microbiota, we performed fecal microbiota transplantation (FMT) from liraglutide-treated donor mice into antibiotic-pretreated CLP recipients. Compared to mice receiving FMT from untreated septic donors (FMT-CLP), those receiving FMT from liraglutide-treated donors (FMT-Lira) showed significantly improved survival (Figure 8A).

Histological analysis revealed reduced lung tissue damage in the FMT-Lira group (Figure 8B), accompanied by decreased serum markers of hepatic and renal injury, including ALT, AST, and creatinine (Figure 8C). Inflammatory cytokine expression in lung tissue, including IL-6, TNF-α, and IL-1β, was also significantly lower in FMT-Lira recipients compared to FMT-CLP controls (Figure 8D).

These findings suggest that liraglutide-induced alterations in the gut microbiota contribute to its protective effects, at least in part, via microbiota-dependent mechanisms.

These results demonstrate that the protective phenotype of liraglutide is transferable via the gut microbiota, establishing the remodeled microbial community as a causal intermediary in the drug’s mechanism of action.

Additionally, we analyzed the citrulline content in fecal samples from Sham, ABX + Lira, and Lira groups. The results demonstrated that the Lira group exhibited the highest citrulline levels, while the ABX + Lira group showed the lowest (Supplementary Figure S3). This indicates that liraglutide promotes citrulline production, and this effect is dependent on the presence of gut microbiota.

### 3.9. Functional and Clinical Validation of Citrulline as a Key Microbial Metabolite

Prompted by its identification as one of the most significantly upregulated metabolites in the liraglutide-remodeled gut metabolome (Section 3.3), we focused on citrulline for functional validation. This amino acid derivative sits at the intersection of microbial and host metabolism and is implicated in immunomodulation and vascular homeostasis—processes central to sepsis pathophysiology.

**Figure 9:**
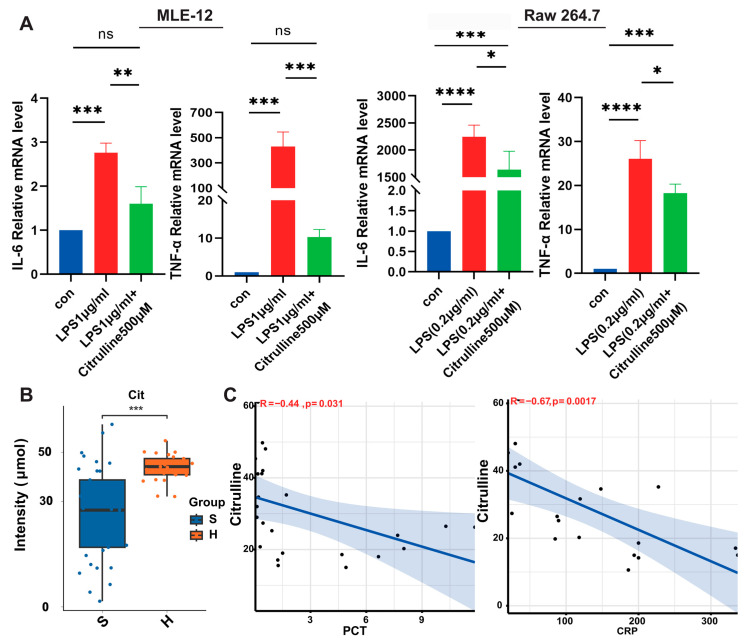
Functional and clinical validation of citrulline. (**A**) The mRNA expression levels of pro-inflammatory cytokines (Tnf-α and Il-6) in MLE-12 cells and RAW264.7 cells after pretreatment with 500 μM citrulline (Cit) or vehicle (Veh) for 4 h, followed by stimulation with LPS (1 µg/mL and 0.2 µg/mL, respectively) for 20 h. Data are presented as the mean ± SEM (*n* = 3 independent experiments). ns, not significant; * *p* < 0.05, ** *p* < 0.01, *** *p* < 0.001, **** *p* < 0.0001 vs. Veh + LPS group (two-tailed Student’s *t*-test). (**B**) Plasma citrulline concentrations in healthy controls (H, *n* = 29) and sepsis patients (S, *n* = 39). (**C**) Correlation analysis between plasma citrulline levels and clinical severity biomarkers (PCT and CRP) in septic patients.

To assess the potential of citrulline in modulating inflammation, we treated MLE-12 and Raw 264.7 cell lines with LPS (1 µg/mL and 0.2 µg/mL, respectively) and evaluated the effects of citrulline (500 µM) on inflammatory cytokine production. In both cell lines, citrulline treatment significantly reduced the expression of IL-6 and TNF-α mRNA levels compared to LPS-only treatment (Figure 9A).

We then translated this finding to the clinical setting. Plasma citrulline levels were significantly lower in septic patients compared to healthy controls (Figure 9B). Furthermore, its concentration exhibited significant inverse correlations with established clinical severity biomarkers, including procalcitonin (PCT; R = −0.67, *p* = 0.0017) and C-reactive protein (CRP; R = −0.44, *p* = 0.031) (Figure 9C). A negative correlation between citrulline and lactate levels was also observed, though it did not reach statistical significance, likely due to the limited sample size (Supplementary Figure S4).

Collectively, these data substantiate citrulline as a functionally active metabolite capable of modulating inflammatory responses. Its depletion in human sepsis and correlation with disease severity underscore its potential clinical relevance, supporting the premise that citrulline may be one operative mediator within the liraglutide-remodeled gut–microbiota–metabolite axis.

## 4. Discussion

Sepsis-induced multiple organ dysfunction remains a leading cause of mortality in intensive care, with current therapies primarily focused on empirical antimicrobials and circulatory support [6,8,9]. However, this approach faces dual challenges: increasing treatment failure due to pathogen heterogeneity and antimicrobial resistance [19], and the treatments themselves exacerbating the patient’s condition by disrupting the microbiota, inducing secondary infections, or causing a “secondary hit” [20]. Consequently, there is an urgent need for novel adjunctive therapies that go beyond mere “antimicrobial” and “pressor” interventions [13,21].

Glucagon-like peptide-1 receptor agonists (GLP-1RAs), originally developed as glucose-lowering agents, have been recognized for their pleiotropic benefits, including anti-inflammatory activity, maintenance of intestinal barrier function, and modulation of gut microbial composition [22]. Prior preclinical studies have reported organ-protective effects of GLP-1RAs in various inflammatory models [23,24,25]. However, to our knowledge, no previous study has systematically examined the impact of liraglutide on the gut microbiota and its metabolic outputs in the context of sepsis.

In our clinical cohort, we observed significantly elevated circulating GLP-1 levels in patients with early-stage sepsis compared to healthy controls (Figure 2A), consistent with prior reports of GLP-1 elevation during inflammatory and metabolic stress, supporting the notion that endogenous GLP-1 is upregulated as an adaptive response to septic physiology [26,27].

Our study addresses this gap by integrating multiple lines of evidence—clinical GLP-1 profiling, a well-established murine CLP model, 16S rRNA sequencing, untargeted fecal metabolomics, microbiota depletion, and fecal microbiota transplantation (FMT)—into a unified analytical framework. Liraglutide administration in septic mice significantly improved survival and reduced systemic pro-inflammatory cytokines. At the molecular level, liraglutide downregulated pro-apoptotic *Caspase-3* and the metabolic regulator Lipin-1, while upregulating anti-apoptotic *BCL2* expression in lung tissues (Figure 2L–N), suggesting a multifaceted protective effect.

In addition to modulating host immune and apoptotic responses, liraglutide markedly influenced the gut microbial ecosystem. Sepsis induced profound dysbiosis, characterized by a loss of diversity and depletion of beneficial taxa, consistent with previous observations in both clinical and preclinical settings [19,20]. The CLP group shows enrichment of *Firmicutes* and *Actinomycetales*. The Lira group is characterized by a distinct *Verrucomicrobia*–*Akkermansia* lineage. The relative abundances of selected genera *Lactobacillus Mucispirillum* were significantly altered in the Lira group compared to the CLP group. Specifically, *Mucispirillum*, a genus often associated with intestinal inflammation and increased gut permeability [28,29], showed a marked decrease in relative abundance following liraglutide administration. The F/B ratio, a commonly used indicator of gut microbial balance that is typically elevated in various inflammatory conditions [30,31], was notably reduced in the Lira group, approaching levels observed in healthy control animals. Liraglutide treatment partially restored microbial diversity and selectively enriched genera such as *Akkermansia*, which has been implicated in maintaining mucosal integrity and regulating host inflammation. Although *Akkermansia* itself has been associated with improved outcomes in sepsis models [32,33], the interactions within the gut microbiota are complex and likely drive metabolic changes through collective activity rather than the action of a single species. Our findings suggest that liraglutide not only alters microbial composition but also functionally reprograms the microbial metabolic output, which has been a pivotal player in modulating host immune responses and systemic inflammation during critical illness [34,35]. The relationship between the gut microbiota and metabolism is increasingly recognized as central to disease progression. Emerging evidence highlights that microbial metabolites play pivotal roles in modulating host immunity and inflammation [36]. GLP-1 and its receptor agonists (GLP-1RAs) can in turn modulate the gut luminal environment, including pH and motility, thereby influencing microbial community composition [37].

Taken together, our study provides integrative evidence that liraglutide ameliorates sepsis not solely through direct immunomodulatory effects, but by reshaping the gut microbial landscape and its metabolic output. Among these downstream metabolites, citrulline emerged as a functionally relevant molecule capable of attenuating inflammatory responses and correlating with disease severity in patients. In contrast to conventional pharmacologic therapies or fecal microbiota transplantation, interventions targeting specific microbial metabolites may offer a more defined, reproducible, and safer means of influencing host–microbiota crosstalk, while minimizing the risks inherent to whole-microbiota or systemic drug-based approaches. The upregulation of citrulline may not stem from a single microbial species but rather reflects the integrated metabolic activity of the reshaped microbial community, shaped by interspecies interactions and host–microbe crosstalk. While our findings do not fully elucidate the mechanistic contributions of individual microbial taxa or metabolites, they establish a coherent framework linking GLP-1RA treatment to improved outcomes via the microbiota–metabolite axis. Further studies focusing on the use of GLP-1RA drugs or targeted metabolic supplementation or synthetic analogs may help to translate these insights into clinically actionable therapies for sepsis.

## 5. Limitations

Several limitations of this study should be acknowledged. First, although the microbiota-dependency of liraglutide’s protective effects was demonstrated through antibiotic ablation and fecal microbiota transplantation (FMT), the precise microbial taxa and molecular pathways mediating these effects remain to be elucidated. Future investigations employing gnotobiotic models, mono-colonization strategies, or high-resolution metagenomic and metabolomic profiling are warranted to define specific microbial contributors and functional mechanisms.

Second, while citrulline was identified as a representative metabolite and functionally validated in vitro, its causal role in mediating liraglutide-induced protection in vivo was not directly demonstrated. Further mechanistic investigations are warranted to elucidate the molecular pathways through which citrulline exerts its immunomodulatory effects and to determine its contribution, combined with pathway-level analyses; this would help to clarify its mechanistic relevance and therapeutic potential.

Third, the animal model used in this study, typically involving young, healthy mice subjected to experimental sepsis, may not fully recapitulate the complexity of human sepsis, which often occurs in elderly or immunocompromised individuals with comorbidities. Additionally, although the liraglutide dosing regimen was based on established pharmacokinetic scaling and prior studies, minor variability in dosing across experimental groups may have introduced confounding effects. Future studies should involve more detailed and carefully designed groupings, including considerations of dosage, administration route, and dosing frequency, to better understand the optimal treatment parameters for liraglutide in sepsis to help minimize this potential variability and better define the exposure–response relationship. The generalizability of our findings to diverse patient populations and clinical settings therefore needs to be evaluated in future translational studies. The clinical component of this study was limited by sample size and heterogeneity, which may constrain the generalizability of the observed associations between circulating citrulline levels and sepsis severity. Larger, multi-center cohorts with longitudinal sampling are needed to confirm these findings and assess their prognostic utility.

Finally, although murine models offer mechanistic insights, interspecies differences in immune response and GLP-1 pharmacodynamics necessitate caution when extrapolating results to human sepsis. The clinical applicability of GLP-1RAs in septic patients, particularly with respect to their gastrointestinal and glycemic effects, requires further evaluation in translational and clinical studies.

In summary, our integrated approach provides preclinical evidence for the gut microbiota-mediated protection of liraglutide. The correlative findings in the human cohort, albeit supportive, are preliminary due to the modest sample size and lack of patient-derived microbial or metabolic profiling. Therefore, the key mechanistic insights generated here require validation in larger, prospective clinical studies and interventional models before any therapeutic application can be considered.

## 6. Conclusions

In summary, this study demonstrates that liraglutide confers protection against sepsis-induced organ dysfunction, an effect closely associated with modulation of the gut microbiota and its metabolic output. Through an integrated approach combining clinical observations, a murine CLP model, 16S rRNA sequencing, untargeted metabolomics, and microbiota-manipulation experiments, we show that liraglutide reshapes microbial composition and enhances the production of beneficial metabolites, particularly citrulline. Functional validation suggests that citrulline may contribute to the anti-inflammatory effects observed in both experimental and clinical settings.

Importantly, our findings underscore the potential of targeting microbial metabolites as a more defined and mechanistically informed approach compared to broader interventions. However, given the exploratory nature of the clinical correlations and the absence of microbiota or metabolomic profiling in patient samples, these observations should be interpreted primarily as mechanistic insights that establish a rationale for future investigation, rather than evidence of immediate translational applicability. Further studies in larger, longitudinal clinical cohorts and interventional models are required to validate the causal role of liraglutide and its associated gut metabolites and to assess the feasibility of metabolite-targeted strategies in human sepsis.

## Data Availability

Publicly available datasets analyzed in this study are available in the GEO database under accession number GSE232753.

## Supplementary Materials

The following supporting information can be downloaded at https://www.mdpi.com/article/10.3390/nu18030531/s1. Supplementary Figure S1. Heatmap of metabolites differentially abundant between CLP and liraglutide-treated (Lira) groups. Supplementary Figure S2. Liraglutide modulates specific metabolites in sepsis. Supplementary Figure S3. Fecal citrulline levels are elevated by liraglutide in a gut microbiota-dependent manner. Supplementary Figure S4. Association of plasma citrulline with lactate.
