# Supplementary figures and images for: GLP-1RA Liraglutide Attenuates Sepsis by Modulating Gut Microbiota and Associated Metabolites
Source: Nutrients. 2026 Feb 5;18(3):531. doi: 10.3390/nu18030531 (PMC12899774; doi:10.3390/nu18030531)

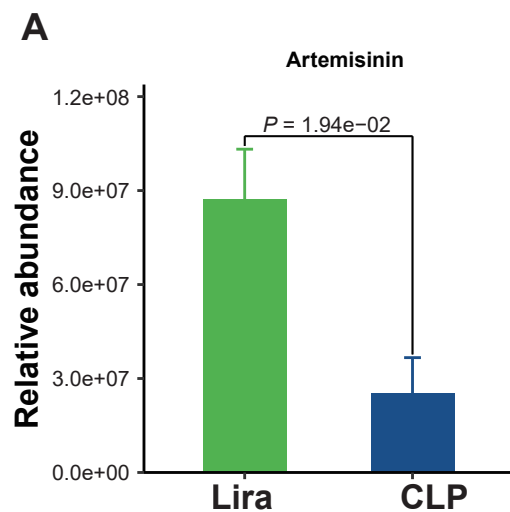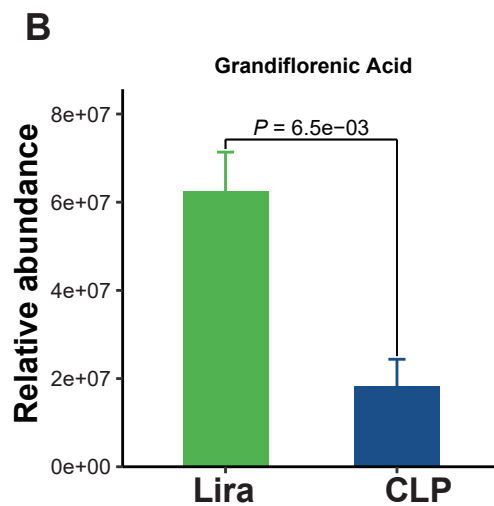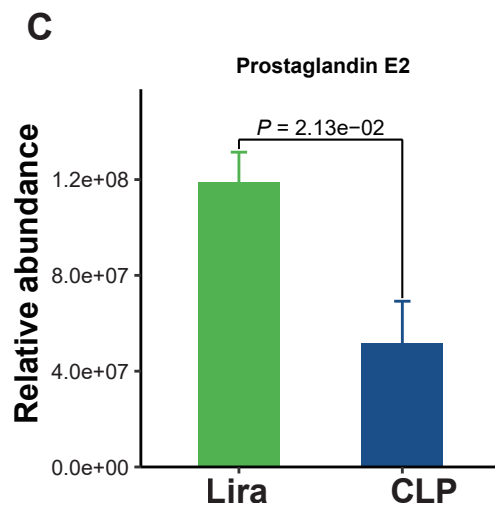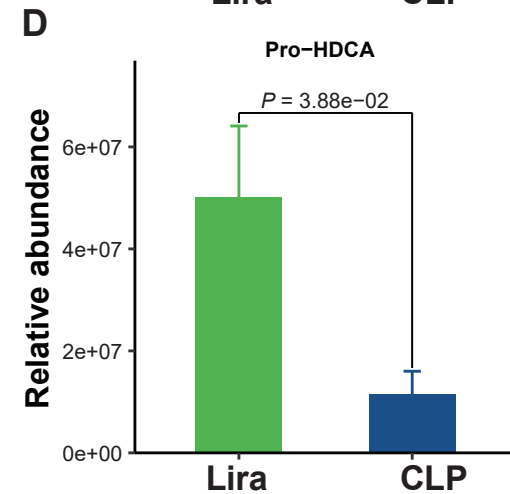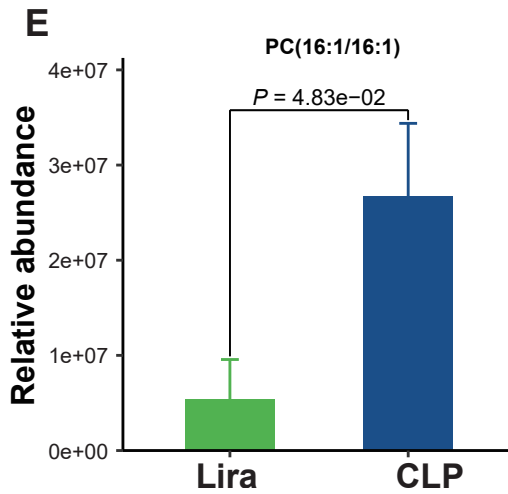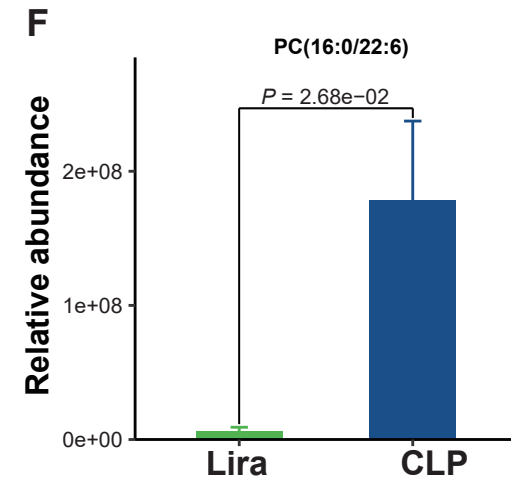

Supplement: Supplementary file 1 [file nutrients-18-00531-s001.zip › supplement figure. s2.pdf]

Sham ABX+Lira Lira

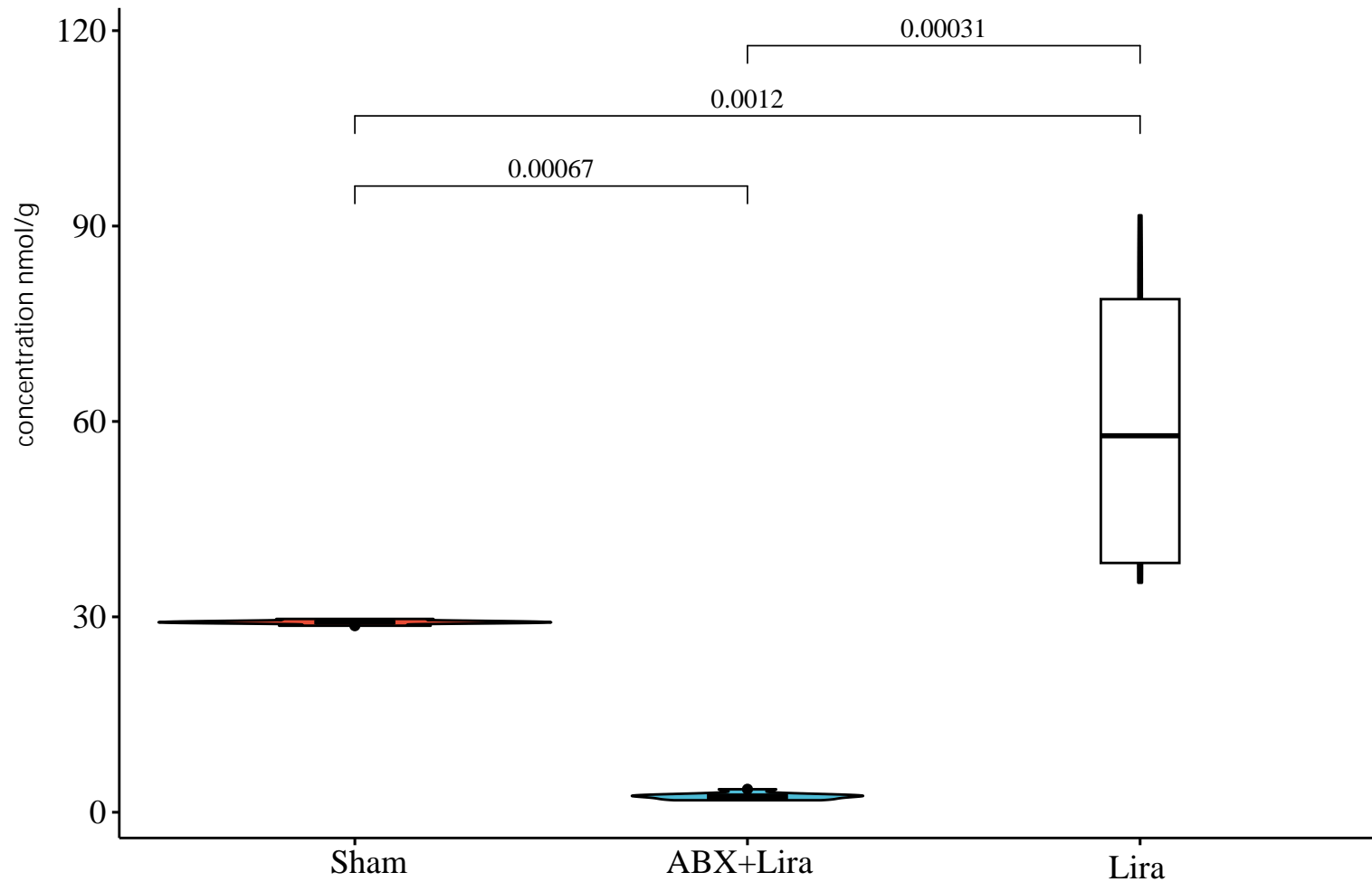

Supplement: Supplementary file 1 [file nutrients-18-00531-s001.zip › Supplementary Fig. S3.pdf]

Scatter plot for Lactate and Citrulline

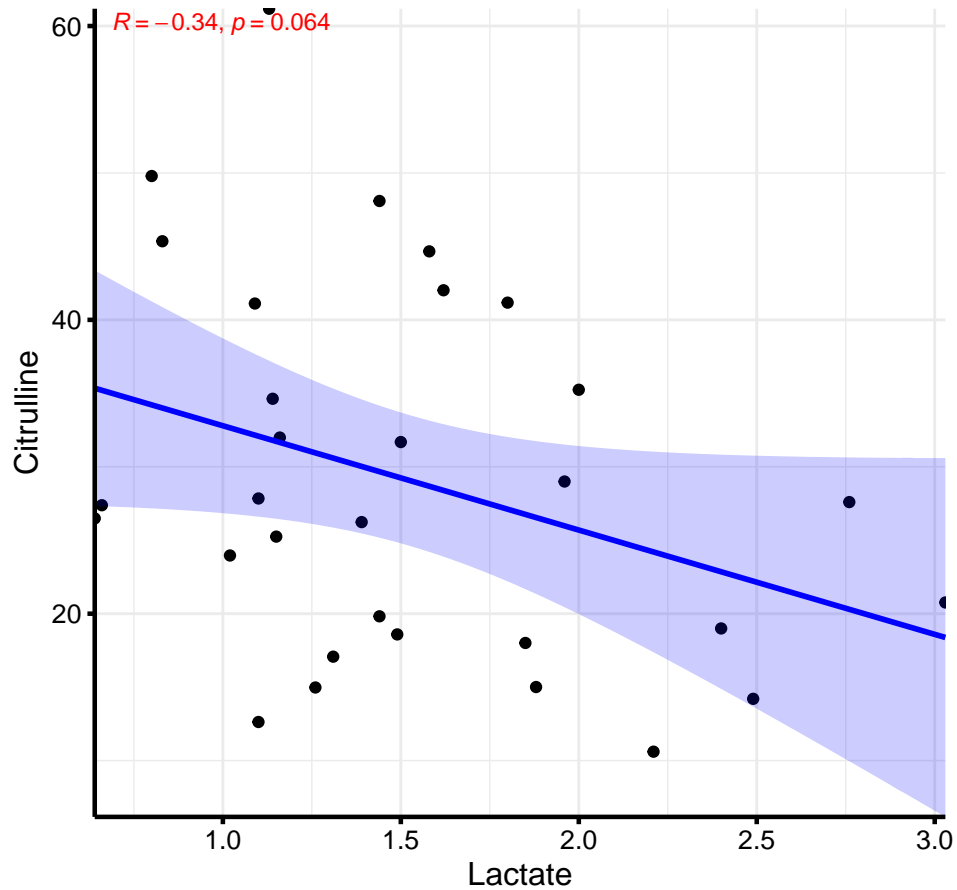

Supplement: Supplementary file 1 [file nutrients-18-00531-s001.zip › Supplementary Fig. S4.pdf]

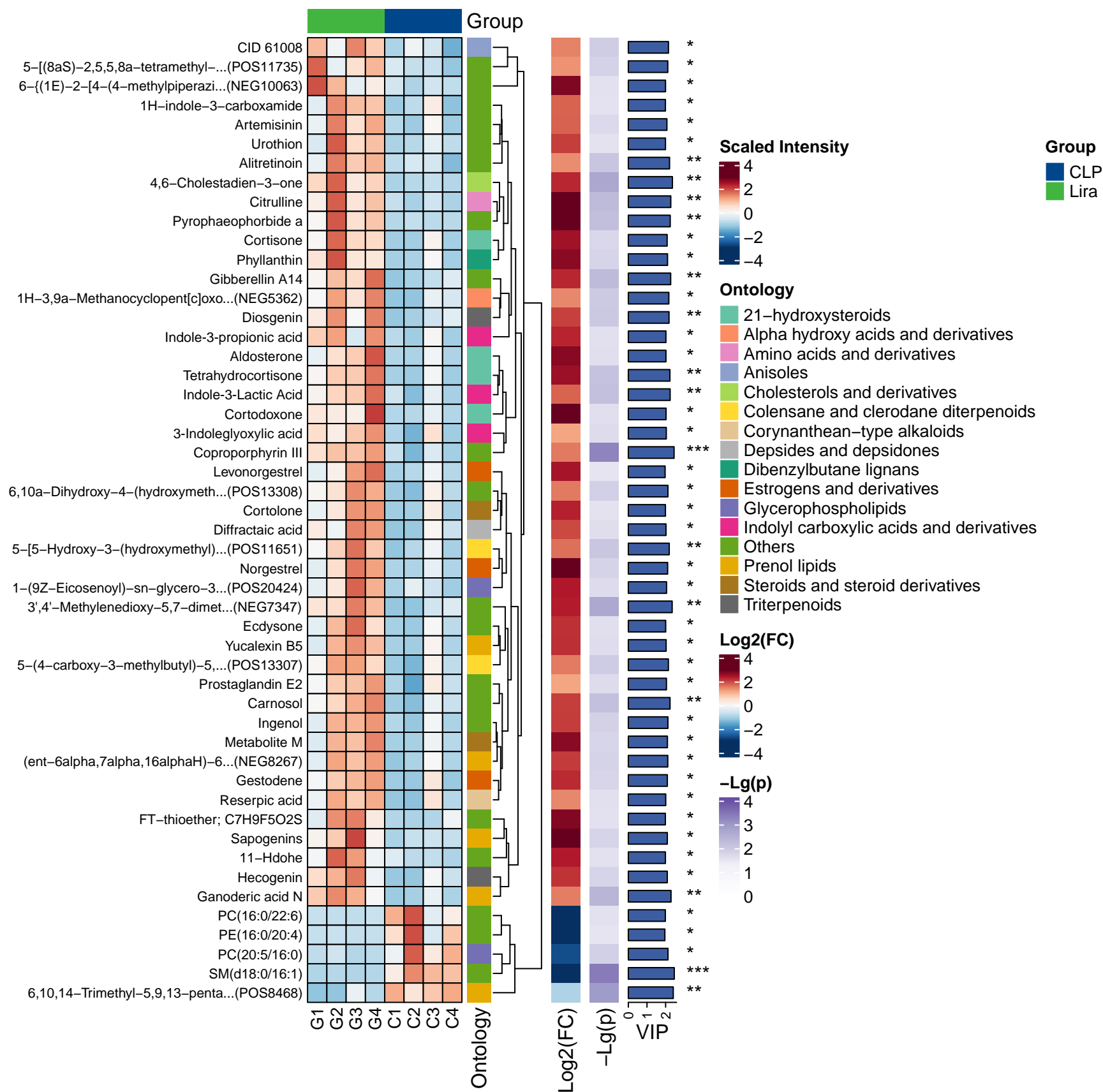

Supplement: Supplementary file 1 [file nutrients-18-00531-s001.zip › Supplementary Figure S1.pdf]
